# Mitochondrial genome of the Common burrowing snake *Achalinus spinalis* (Reptilia: Xenodermatidae)

**DOI:** 10.1080/23802359.2017.1365643

**Published:** 2017-08-22

**Authors:** Lifang Peng, Diancheng Yang, Shuangquan Duan, Song Huang

**Affiliations:** a School of Sciences, Tibet University, Lhasa, China;; b College of Life and Environment Sciences, Huangshan University, Huangshan, China

**Keywords:** Xenodermatidae, *Achalinus spinalis*, mitochondrial genome

## Abstract

Common burrowing snake *Achalinus spinalis* is the type species of *Achalinus*. The complete mitochondrial genome (mitogenome) sequence of *A. spinalis* was determined by using a PCR-based method. The total length of mitogenome is 17,165 bp and contains 13 protein-coding genes, 22 tRNA genes, 2 ribosome RNA genes and 2 control regions (CR). All the protein-coding genes in *A. spinalis* were distributed on the H-strand, except for the ND6 subunit gene and eight tRNA genes which were encoded on the L-strand. The phylogenetic tree of *A. spinalis* and 12 other closely species was built. The DNA data presented here will be useful to study the evolutionary relationships and genetic diversity of *A. spinalis*.

The Common burrowing snake *Achalinus spinalis*, belonging to the family Xenodermatidae, is a widely distributed species in China, Japan and Northern Vietnam (Orlov et al. 2000; Zhao 2006). Previously, the genus *Achalinus* (Peters 1869) was put in Xenodermatinae in Colubridae (Peters 1869; Zhao et al. 1998). Recent phylogenetic studies supported the Xenodermatid snakes as a distinctly familial status (Vidal et al. 2007; Zaher et al. 2009; Pyron et al. 2013). Although there are nine species in the genus *Achalinus* presently, a few sequences of this genus have been determined. Two of these sequences are the complete mitochondrial genome of *Achalinus meiguensis* (Wang et al. 2009) and *Achalinus rufescens* (Zhang et al. 2017). In this article, we determined and described the mitogenome of *A. spinalis* in order to obtain basic genetic information about this species.

The specimen of *A. spinalis* (Voucher number: HS12093) was collected from Taibai County, Shaanxi Province, China (34°01.861′N, 107°13.686′E). It was deposited in the Museum of Huangshan University (Voucher number: HUM201200001). The complete mitogenome of *A. spinalis* (GenBank accession number KT897594) was sequenced to be 17,165 bp which consisted of 13 typical vertebrate protein-coding genes, 22 transfer RNA (tRNA) genes, 2 ribosomal RNA (rRNA) genes and 2 D-loop, which is similar to the typical mtDNA of snakes and other vertebrates (Boore 1999; Sorenson et al. 1999). The overall base composition of the entire genome was as follows: A (30.9%), T (24.8%), C (30.7%) and G (13.6%), which the percentage of A + T (55.7%) reflected a typical sequence feature of the vertebrate mitogenome. Most of the *A. spinalis* mitochondrial genes are encoded on the H-strand except for the ND6 gene and eight tRNA genes, which are encoded on the L-strand. The positions of RNA genes were predicted by the MITOS (Bernt et al. 2012), and the locations of protein-coding genes were identified by comparing with the homologous genes of other closely related species. Among the mitochondrial protein-coding genes, the ATP8 was the shortest, while the ND5 was the longest. Seven of the 13 protein-coding genes (COII, ATPase 6, COIII, ND4L, ND4, ND5 and CYT b) initiate with ATG as start codon, while COI and ATPase eight genes are initiated by GTG, ND3 gene begin with ATT and the other protein-coding genes used ATA as start codon. Seven protein-coding genes end with complete stop codons (AGG, TAA and AGA), and the other six genes end with T as the incomplete stop codons, which were presumably completed as TAA by post-transcriptional polyadenylation (Anderson et al. 1981). The 22 tRNA genes range in size from 51 to 74 bp. The 12S rRNA (921 bp) and 16S rRNA (1481 bp) are located between the tRNA-Phe and ND1 genes and separated by the tRNA-Val gene. The control region 1 (CR1) of the *A. spinalis* mitogenome in size is 1026 bp and was located between the tRNA-Ile and tRNA-Leu genes, while the control region 2 (CR2) is 1064 bp long, between the tRNA-Pro and tRNA-Phe genes.

To further validate the new determined sequences, we selected the 12 protein-coding genes located on heavy strand except for ND6 which encoded on the light strand of *A. spinalis* in this study and together with other 12 closely related species from GenBank to perform phylogenetic analysis. These species were as follows: *A*. *meiguensis*, *Cylindrophis ruffus*, *Deinagkistrodon acutus*, *Crotalus horridus*, *Ovophis okinavensis*, *Protobothrops dabieshanensis*, *P*. *jerdonii, Python regius*, *Viridovipera stejnegeri*, *Cryptelytrops albolabris* and *Charina trivirgata*. A maximum-likelihood (ML) tree was constructed based on the dataset by online tool RAxML (Stamatakis et al. 2008) (Figure 1). The phylogenetic analysis result was consistent with the previous research. It indicated that our new determined mitogenome sequences could meet the demands and explain some evolution issues.

**Figure 1.**
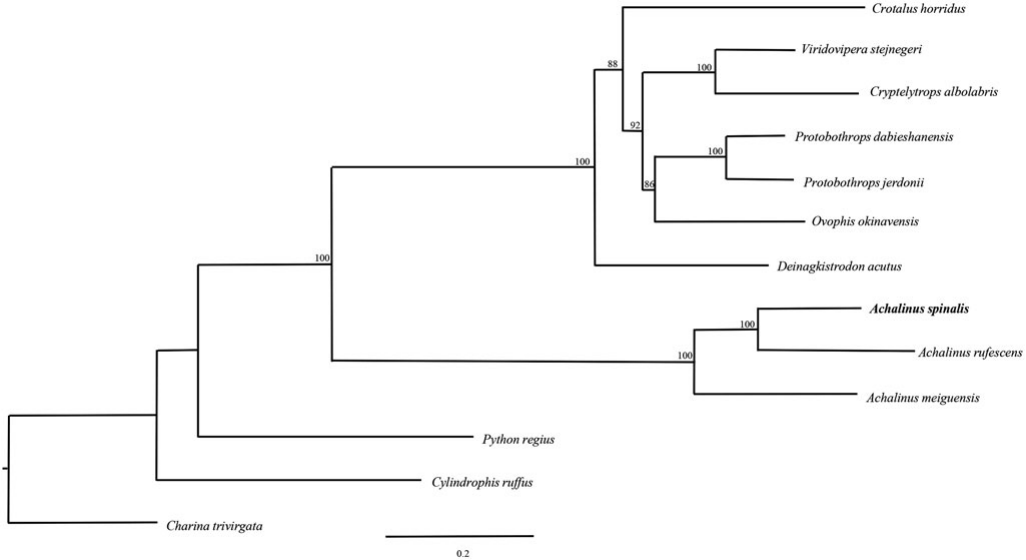
A maximum-likelihood (ML) tree of *A. spinalis* in this study and other 12 closely related species was constructed based on the dataset of 12 concatenated mitochondrial protein-coding genes by online tool RAxML. The numbers above the branch meant bootstrap value. Bold black branches highlighted the study species and corresponding phylogenetic classification. The analyzed species and corresponding NCBI accession numbers are as follows: *Crotalus horridus* (HM641837), *Viridovipera stejnegeri* (FJ752492), *Cryptelytrops albolabris* (KF311102), *Protobothrops dabieshanensis* (KF003004), *P. jerdonii* (KC112560), *Ovophis okinavensis* (AB175670), *Deinagkistrodon acutus* (EU913476), *A. spinalis* (FJ424614), *Achalinus rufescens* (KT897595), *A*chalinus *meiguensis* (FJ424614), *Python regius* (AB177878), *Cylindrophis ruffus* (AB179619) and *Charina trivirgata* (GQ200595).
